# 279. Inhaled Sargramostim (rhu GM-CSF) Leads to Enhanced SARS-CoV-2 Virus-Specific Immune Response and Viral Clearance: Results of the Biomarker Cohort of a Randomized, Double-Blind, Placebo-Controlled Phase 2b Trial in Non-Hospitalized Patients with COVID-19

**DOI:** 10.1093/ofid/ofad500.351

**Published:** 2023-11-27

**Authors:** Ila Joshi, Fiona Garner, Debasish F Roychowdhury, Lorinda Simms, Sanjeev Ahuja, John L McManus, Edwin P Rock, Rodolfo Perez, Sandro Bacchelli, Robert Paine

**Affiliations:** Partner Therapeutics, Inc., Lexington, Massachusetts; Partner Therapeutics, Inc., Lexington, Massachusetts; Partner Therapeutics, Inc., Lexington, Massachusetts; Partner Therapeutics, Inc., Lexington, Massachusetts; Partner Therapeutics, Inc., Lexington, Massachusetts; Partner Therapeutics, Inc., Lexington, Massachusetts; Partner Therapeutics, Inc., Lexington, Massachusetts; Encore Medical Research, Hollywood, Florida; Encore Medical Research, Hollywood, Florida; University of Utah, Salt Lake City, Utah

## Abstract

**Background:**

Progression from mild to severe SARS-CoV-2 infection correlates with humoral and cellular immune signatures that adjust to a multiplying viral load. Enhancing immunity with host-directed therapy, such as sargramostim (yeast-derived, recombinant human granulocyte-macrophage colony-stimulating factor [GM-CSF]), may prevent disease progression and reduce severity.

**Methods:**

This prospective, randomized, double-blind, placebo-controlled study enrolled symptomatic vaccinated and unvaccinated non-hospitalized patients with mild/moderate COVID-19 at high risk for progression. Patients received daily sargramostim 250 mcg or placebo, inhaled via nebulizer, for 5 days. Figure 1 lists study endpoints.

**Figure:**
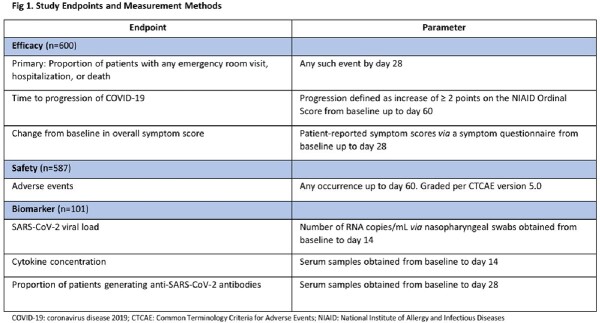

**Results:**

From April 28, 2021 to January 31, 2022, 101 patients consented and provided samples for the biomarker cohort (Fig 2).

No difference was seen in the overall study primary endpoint (n=21/301 sargramostim, n=16/299 placebo, p=0.4). Treatment-emergent adverse events were similar in both arms. In the biomarker cohort, SARS-CoV-2 viral clearance by day 14 was enhanced on the sargramostim arm (p=0.0064, Fig 3). Virus was undetectable in a larger proportion on the sargramostim arm. Sargramostim reduced viral load more in vaccinated than unvaccinated patients. Inflammatory cytokine levels did not increase with treatment.

Kinetics and magnitude of antibody response against SARS-CoV-2 antigens differed by group. After an initial IgM-titer peak at day 14, IgM titers at day 28 were lower in the vaccinated-sargramostim arm than the vaccinated-placebo arm (Fig 4A). The unvaccinated-sargramostim arm maintained higher IgM titers than the unvaccinated-placebo arm. Overall IgG titers were higher for vaccinated patients across both treatment arms. However, the vaccinated-sargramostim arm had higher IgG4 titers associated with IgG4-isotype-class switching than the vaccinated-placebo arm (Fig 4B).

**Figure:**
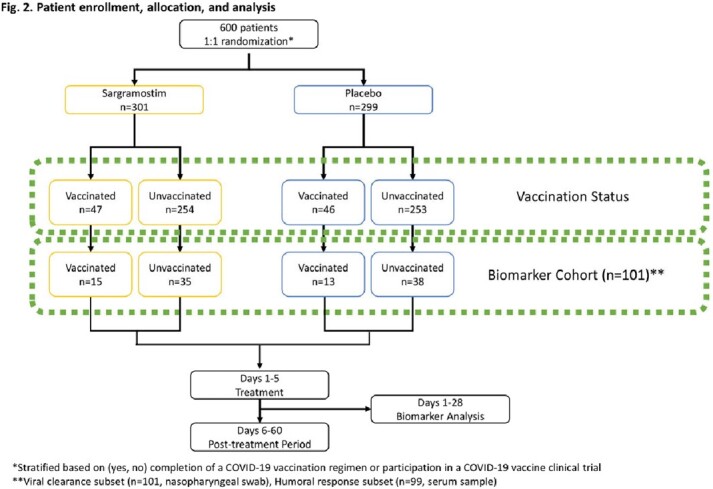

**Figure:**
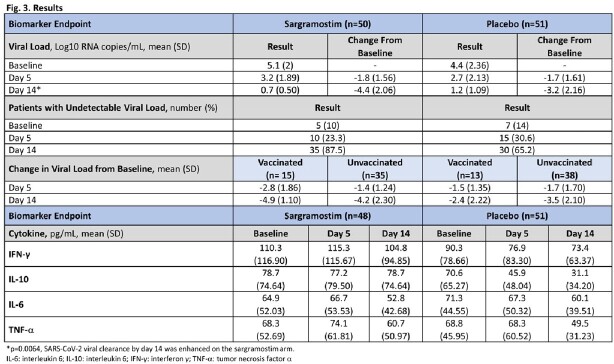

**Figure:**
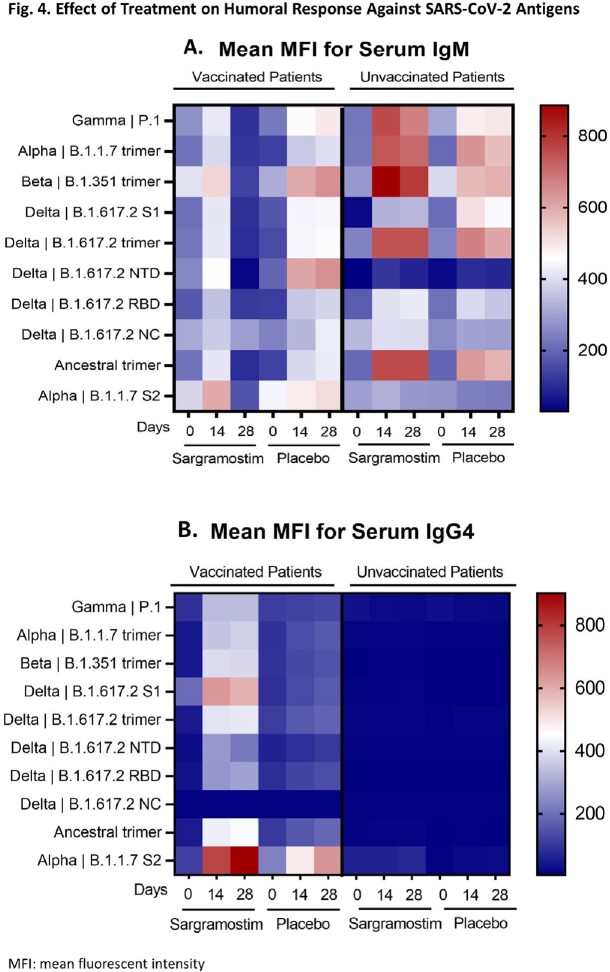

**Conclusion:**

The primary clinical endpoint was not met. Biomarker analyses suggest inhaled sargramostim reduces SARS-CoV-2 viral load and alters humoral kinetics and expression, more so in those vaccinated. Results suggest the potential of sargramostim as a virus agnostic, host-directed immunomodulator.

**Disclosures:**

**Ila Joshi, PhD**, Partner Therapeutics, Inc.: Patents|Partner Therapeutics, Inc.: Salary|Partner Therapeutics, Inc.: Stocks/Bonds **Fiona Garner, PhD**, Partner Therapeutics, Inc.: Employment|Partner Therapeutics, Inc.: Stocks/Bonds **Debasish F. Roychowdhury, MD**, Partner Therapeutics, Inc.: Employment|Partner Therapeutics, Inc.: Ownership Interest|Partner Therapeutics, Inc.: Stocks/Bonds **Lorinda Simms, MSc**, Partner Therapeutics, Inc.: Advisor/Consultant|Partner Therapeutics, Inc.: Stocks/Bonds **Sanjeev Ahuja, MD**, Partner Therapeutics, Inc.: Employment|Partner Therapeutics, Inc.: Stocks/Bonds|Scioto Biosciences: Past Employee **John L. McManus, n/a**, JPEO-CBRND's Joint Project Manager for Chemical, Biological, Radiological, and Nuclear Medical: Project agreement MCDC2006-012. Included references to commercial products do not constitute an endorsement by the US DoD or the JPEO-CBRND.|Partner Therapeutics, Inc.: Employment|Partner Therapeutics, Inc.: Stocks/Bonds **Edwin P. Rock, MD, PhD**, Partner Therapeutics: Employment **Robert Paine III, MD**, Partner Therapeutics, Inc.: Advisor/Consultant

